# Who am I now? A scoping review on identity changes in post-stroke aphasia

**DOI:** 10.1080/09638288.2024.2367606

**Published:** 2024-06-19

**Authors:** Rianne Brinkman, Karin Neijenhuis, Mieke Cardol, Carlo Leget

**Affiliations:** a Research Center Innovations in Care, Rotterdam University of Applied Sciences, Rotterdam, the Netherlands; b Care Ethics, University of Humanistic Studies, Utrecht, the Netherlands; c Research Center Healthy Ageing and Allied Nursing, Hanze University of Applied Sciences, Groningen, the Netherlands

**Keywords:** Stroke, aphasia, identity, narrative, healthcare professionals, communication

## Abstract

**Purpose:**

Provide an overview of existing knowledge on the concept of identity and identity changes and formation of people with language disorders of aphasia. To date, there has been limited exploration of identity changes of people with aphasia as communication difficulties have been perceived as a barrier to participation by researchers.

**Method:**

A scoping review was performed utilizing Arksey and O’Malley’s methodological framework. The databases PubMed, PsycINFO, CINAHL, and Cochrane Library were searched, and both descriptive and thematic analysis were conducted.

**Results:**

The search yielded 492 records, of which 20 studies were included. The analysis revealed various theories and definitions of identity. There was limited uniformity in defining identity in the publications. A recurrent theme was, that identity is a social construct and storytelling is the vehicle through which it is navigated. Language and communication are essential in this process.

**Conclusions:**

The model of Narrative Identity might be useful to clarify identity and its dynamic nature, since it integrates various theories of identity referred to in the publications. Different aspects of identity should be considered in future research to add to existing knowledge of identity changes and formation in people with aphasia and to tailor future interventions if necessary.

## IMPLICATIONS FOR REHABILITATION

- Identity formation is a complex and ongoing process for people with post-stroke aphasia, significantly influenced by language disorders. Continuous attention to this process is essential during rehabilitation.
- Effective interventions include peer support through aphasia groups and elicitation of personal narratives, prominently through Guided Self-Determination, Biographic Narrative Intervention, and the My Story project.
- People with aphasia exhibit diverse experiences, encompassing internal variation and ambiguity within themselves, while concurrently navigating dilemmas throughout the process of identity formation.
- The “Narrative Identity Model” might be useful to comprehend identity and its dynamic nature, since it integrates various theories of identity referenced in publications about identity changes in people with aphasia.

## Introduction

A sudden event like a stroke shifts a person’s life story and causes identity changes. Aphasia is a language disorder that affects the ability to communicate and occurs in approximately 30% of all stroke survivors [1]. Communication is crucial in identity formation. Thus, for people with aphasia (PWA), there is an even greater challenge to reshape identity [2]. This review focuses on the published literature about identity formation in PWA.

### Theories of identity formation

In the literature different definitions are used to define the concept of identity. Moreover, the terms “identity” and “self” are often used interchangeably. Since the narrative turn in the social sciences in the 1970s and 1980s, identity has often been explained in the literature from the perspective of social constructionism. Social constructionism assumes that our knowledge of the world, including the understanding of human beings, is a product of human thought rather than rooted in an observable, external reality [3]. The French philosopher Paul Ricoeur [4] has had a major influence on this narrative turn in the humanities [5]. According to Ricoeur [4], people construct an understanding of who they are by giving meaning to the stories they exchange with others. This is what he calls narrative identity. Narrative identity is formed relationally and situationally in the present (synchronic) and evolves through time (diachronic) [4]. Identity is shaped by creating narratives mediated through social interaction. Accordingly, the concept of identity is strongly interrelated with narrative [2].

Also introduced in the 1970s and 1980s by psychologists Tajfel and Turner [6] and consistent with social constructionism is social identity theory. This theory is frequently used in studies on stroke and identity changes to understand identity formation. Social identity theory is based on the concept that identity is shaped through group membership [6]. The social groups to which an individual belongs can shape the understanding of self by being internalized, thereby influencing identity formation.

This means that social categories such as gender, health condition, or disability partially define identity. The sudden onset of a health condition such as stroke, during which an individual may lose social identities (for instance, being an able-bodied person), can threaten identity formation [7]. For PWA, identity formation becomes even more threatened as a consequence of their communicative impairments.

In the field of aphasia and identity formation, Barbara Shadden substantially influenced the current theoretical background for understanding identity formation in PWA. She and her colleagues introduced the Four-Domain Interdisciplinary Framework [8] designed to discuss the narrative self after the onset of neurogenic communication disorders such as aphasia. To the best of our knowledge, this is the only interdisciplinary framework to consider identity changes after stroke and aphasia. This framework addresses four dimensions of everyday life (cultural aspect, roles, situational interactions, biography) as well as the underlying concepts from which the self is constructed (agency and power), and provides professionals with consistent, universal terminology to discuss identity. This framework was also established from the perspective of social constructionism, such as Ricoeur’s narrative identity theory and Tajfel and Turner’s social identity theory, but specifically focused on people with communication disorders. Therefore, we use the Four-Domain Interdisciplinary Framework [8] as a foundation for reviewing the literature on aphasia and identity.

Language disorders have an enormous impact on identity and identity formation [2,8,9], since narrative identity is formed through the stories we share with others. Storytelling is a complex process that requires cognitive, language, and motor skills [8]. Furthermore, people need the capacity to interpret and employ different nonverbal behaviors and the awareness of self and others in order to choose what will be shared with whom under what conditions.

### Research on identity changes after stroke

Considerable research has been performed on identity changes after stroke. Important stroke-related themes that emerged in these studies were change of self-perception in relation to the pre-stroke self, coping with a new life and disability [10–14], a disruption of body and self [11,14], and a change of meaning in life [12]. Stroke survivors viewed themselves as significantly less independent, satisfied, and active [10]. Many survivors lead more home-centered lives with reduced social relationships and are less actively involved in their communities [10–12,14].

However, it should be mentioned that PWA are often excluded from identity-related research because of their communicative impairments. Inclusion criteria often impose high demands on communicative ability of the participants. In some studies, communicative impairments are not mentioned [9]. Not all studies exclude individuals with aphasia. In the studies performed by Kitzmüller [13] and Pallesen [14], 20 to 45% of participants had aphasia. Yet, the analysis did not differentiate between experiences of PWA and stroke survivors without aphasia. Ultimately, studies on identity changes after stroke are biased toward stroke-survivors who were able to share their stories. PWA encounter additional obstacles to reshaping their identity compared to people with stroke without aphasia due to their communication difficulties. Therefore, more knowledge about the precise impact of language disorders on identity formation of PWA is required. The importance of integrating identity-related issues into the study of PWA is underlined by several researchers [8,9,15]. In the existing literature, regarding the experiences of PWA concerning identity changes and identity formation, various terms are used to define identity and salient themes. Greater knowledge and consistency of the concept of identity are required in order to compare studies or build on existing knowledge.

### Changes in psychosocial functioning in aphasia

A better understanding of identity changes in aphasia is urgently required because a threatened identity is considered one of several psychosocial challenges faced by PWA [16,17]. PWA are especially susceptible to changes in psychosocial functioning because of their communicative difficulties. In addition to identity changes, PWA experience reduced social interaction, social isolation, unemployment and withdrawal from leisure activities [16,17]. Furthermore, suicide risk after stroke is reported to be as high as 73% [18] and a large number of people with stroke experience anxiety (32%) [19] and depression (23%) [20]. Alarmingly, the prevalence of anxiety and depression among individuals with aphasia following a stroke is higher compared to those without aphasia. Among individuals with aphasia, 44% experience anxiety [19], and they are 7.5 times more likely to develop depression than stroke survivors without aphasia [20]. Changes to mental health and wellbeing extend beyond the PWA, with family members experiencing depression at prevalence rates of 46% [21].

These psychosocial issues have a distinct effect on recovery, the psychosocial adjustment process, and the reaction to rehabilitation [16,22]. Consequently, the need for appropriate mental health services is substantial in PWA. In addition, several stroke clinical guidelines emphasize the need for general psychological care for PWA, (i.e., monitoring and assessment of emotional needs with validated instruments and triage to psychological care tailored to the person’s needs if required [23–26]). Stepped psychological care can include various levels of support, such as mental healthcare for moderate to severe mental illness, to less intensive psychological care such as peer support through aphasia groups and counseling [27]. PWA express great need for positivity, supported communication, and access to customized therapy [28]. Nevertheless, they are currently attempting to navigate their way through communication and mood problems with limited psychological support and services in stroke rehabilitation.

### Healthcare and support of identity formation

Healthcare professionals agree on the value of focusing on psychological care, including support of identity formation in PWA. Although they believe they are in a position to contribute to identity support, healthcare professionals experience barriers to addressing identity issues. They state that they do not possess the knowledge or skills to conduct such activities [2]. Within healthcare, support of identity formation is often referred to specialists like clinical psychologists because it is considered to be beyond the area of expertise of most healthcare professionals [29]. Furthermore, healthcare professionals state that they lack understanding of aphasia and how to support communication, and also that consultations with PWA are time consuming [30]. However, the stepped psychological care model indicates that at level one (subthreshold problems) and level two (mild and moderate problems) therapy can be provided effectively by both psychologists and other healthcare professionals specializing in stroke. Not until level three (severe and persistent disorders of mood) is psychological care restricted to clinical (neuro)psychology and/or psychiatry [27]. While psychologists and psychiatrists have professional expertise in the management of severe mood disorders, skill in supportive communication is of major value for PWA. They require support from their communication partner to formulate their stories [2,8], and they miss the narrative skills to do so without help [31]. In this regard, speech-language pathologists can play an essential role since they are uniquely equipped to facilitate PWA to share their stories [2]. Healthcare could be optimized if there was more knowledge about support of identity formation and communication of PWA across all disciplines.

Conclusively, aphasia may profoundly affect identity formation. Aphasia management could be optimized if there was increased knowledge about identity changes of PWA. This scoping review attempts to fill this gap by evaluating how the concept of identity, identity changes and identity formation is addressed in the existing literature concerning PWA. The goal is not only to provide a comprehensive overview of existing knowledge on identity and aphasia, but also to clarify how these findings can inform clinical practice.

## Methods

A scoping review was conducted to explore the literature on identity and aphasia. Scoping reviews are appropriate for examining broad topics and mapping key concepts [32]. Furthermore, research gaps in the existing literature can be identified. The terminology and methodology used was largely consistent with the PRISMA statement extension for scoping reviews [33,34]. In the final stage of the review process, we consciously diverged from PRISMA terminology. We used the term “reports” rather than “studies” in the final results. This decision was motivated by the fact that the inclusion criteria for this search were broad and included works such as book chapters in addition to peer-reviewed research. The methods used in the current scoping review will be explained according to the five stages of the methodological framework of Arksey and O’Malley [32].

### Stage 1: identifying the research question

The research question for this review was: What knowledge is available in the literature on the concept of identity, identity changes and identity formation of people with post-stroke aphasia and how do they integrate these changes in their lives?

### Stage 2: identifying relevant studies

Published scientific literature was searched on 19 October 2023 via electronic scientific databases (PubMed, PsycINFO, CINAHL and the Cochrane Library). Additionally, the yielded articles’ reference lists were searched to obtain a comprehensive set of literature on this topic. Multiple databases were searched given the nature of the topic and its applicability to diverse disciplines. The research team developed a list of search terms based on orientation searches, which led to search outcomes that best fit the research question. A library expert was consulted for advice on search strategies in the electronic databases. A strategy for searching PubMed was used as the main protocol and modified for other databases.

Since various terms were used to define identity and salient themes, we chose to conduct a broad search by using different keywords to find all studies in which identity changes might be discussed. Therefore, all studies on “life change events” and “personal experiences” were included because possible consequences may have been described in relation to identity. We used “personal narratives” as a keyword to do justice to the interrelationship of narrative and identity [2]. The databases were searched using a search string consisting of two parts and five keywords (Table 1). Medical Subject Headings (MeSH) were used when applicable. Databases were also searched by utilizing Title Abstract terms (TiAb) to find articles not yet indexed with MeSH in the databases. No publication date restriction or other filters were used in the search, ensuring no relevant results were missed.

**Table 1.** Strategy for searching PubMed.

Search string used in PubMed
(“Social Identification”[Mesh] OR “Social ident*”[tiab] OR “Personal Narratives as Topic”[Mesh] OR “Personal Narratives as Topic”[tiab] OR “Personal Narratives”[tiab] OR “Narration”[Mesh] OR “Narration”[tiab] OR “Biographies as Topic”[Mesh] OR “Biograph*”[tiab] OR “experiential knowledge”[tiab] OR “Life Change Events”[Mesh] OR “Life Change Events”[tiab]) AND (“Aphasia”[Mesh] OR “Aphasia”[tiab])
**Keywords**
Identity; personal narratives; experiential knowledge; life change events; aphasia

### Stage 3: study selection

In the study selection phase, the relevance of the records was assessed in two steps: title and abstract level, and full text level. In these two selection steps, the first author (RB) assessed all the records, the co-authors (CL, KN, MC) assessed one third of the records independently of RB. Predefined inclusion criteria at the title and abstract level were: (a) PWA as the target group, (b) personal narratives, identity, and dimensions of the self as a main theme, (c) experiential knowledge and life change events as sub-themes and (d) publications written in Dutch or English. During the title and abstract selection step a four-point scale was used (0 = irrelevant; 1 = possibly relevant; 2 = partially relevant as the sub-themes of criterion a and c had been met; 3 = relevant as criterion a and b had been met). Discussion took place and consensus was pursued when scores between the researchers differed. All records with scores of 2 or 3 were included in the sample. Subsequently, any uncertainties to this point in the review process had not precluded records for assessment at the full text level.

During the title and abstract selection step, the concept of identity was taken broadly to ensure that no records would be inadvertently missed. During the full text selection step, the same procedure was followed, but the inclusion criteria for this level were further specified. To do so, a small subset of four reports was selected as idealized examples. In this way, a demarcated selection was performed to further specify inclusion criteria. The demarcated selection consisted of reports where the research frame focused specifically on identity and/or narrative, and the concept of identity was operationalized in the theoretical framework or elaborated on in the discussion. The final inclusion criteria were: (a) reports in which identity was the aim of research and/or operationalized in the theoretical framework, (b) reports with an open frame focused on “experiences” in which identity was one of the outcomes and was elaborated in the discussion, (c) reports on the dimensions of everyday life and underlying concepts of the self, discussed in relation to identity, and (d) narrative reports discussed in relation to identity. Only peer-reviewed academic publications and books published by academic publishers were included in the sample; therefore, we chose to exclude experiential stories written by PWA. The inclusion of reports was not limited by the design and/or methodological quality of the research. A three-point scale was used during the full text selection step (0 = irrelevant; 1 = possibly relevant; 2 = relevant as all inclusion criteria had been met). Each report was discussed by the first author and one of the co-authors to decide upon inclusion or exclusion. Inconsistencies and uncertainties were discussed within a research team meeting with all four researchers, who also checked the report for the selection criteria. The reference lists of the reports included in this third step were checked for additional relevant reports. This yielded two additional reports.

### Stage 4: charting the data

After the final full text inclusion, a data extraction form was developed based on all full texts and a research team consultation. This data extraction form consisted of descriptive elements (author, year, study aim, theory of identity, study design, data collection, data analysis, study population, age, months post onset, and findings). The first author (RB) charted the data. The co-authors (CL, KN, MC) each assessed one third of the results independently of RB. Subsequently, patterns and inconsistencies were discussed in a research team consultation.

### Stage 5: collating, summarizing, and reporting the results

A summary was made of the main characteristics of the reports, using the descriptive elements of the data extraction form. Thereafter, the researchers conducted a numeric [31] and thematic analysis [35] of the information in the reports. The numerical analysis captured the prevailing areas of research with respect to geographical location, research design [31] and characteristics of the study population regarding age, gender and months post onset. The first phase of the thematic analysis focused on obtaining an overview of data coverage to become familiar with the data [36]. The first author (RB) read all reports; the other three researchers (CL, KN, MC) read one third of the reports independently of RB. Text fragments related to the research question of the scoping review were highlighted and coded using free-line-coding [35]. The coded excerpts from the reports that contained relevant qualitative information about identity and aphasia were independently placed in an Excel file. Based on discussion, a final Excel file including all relevant codes was developed. In the second phase, the codes were independently sorted into related areas by the research team and descriptive themes were named that were close to the findings in the primary reports. The third phase focused on generating new interpretive constructs and explanations by developing analytic themes [35].

## Results

**Figure 1.**
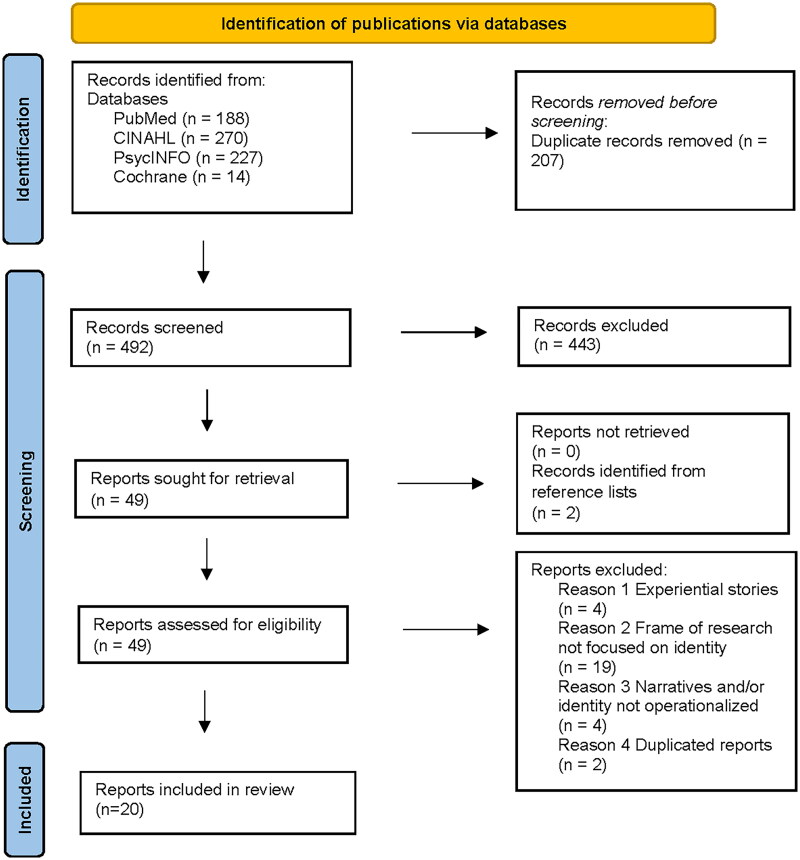
Number of publications included in each step of the review. Based on the PRISMA 2020 flow diagram [37].

Based on the initial 492 records in the databases, 49 reports were included in the full text phase and assessed for eligibility. Reports were excluded when they involved experiential stories written by PWA, the frame of research was not focused on identity, or narratives and/or identity were not operationalized in the theoretical framework. Also, duplicated reports published in two separate publications were excluded for analysis. Eventually, 20 reports were included in this review (Figure 1), published between 1964 and 2023.

### Summary of the reports included in the review

The final set of reports included 3 book chapters published by academic publishers, 5 theoretical papers, and 12 empirical papers from peer-reviewed journals. Detailed descriptions of these reports can be found in Table A1 (Appendix A), which lists the authors, year, country, study aim, design, population, and findings, organized by year to illustrate the evolution of the research field. Additionally, Table A2 (Appendix A) offers a descriptive summary of the theories used in the reports, outlining definitions and theoretical premises of identity.

#### Book chapters

The three book chapters represent the work of two author groups, Shadden and colleagues [8], as well as Pound and colleagues [38] ranging in dates from 2008 to 2018. Shadden et al. [8] focus on adapting narrative theory to aphasia. In general, these two chapters explain how the Four-Domain Interdisciplinary Framework designed to discuss the narrative self can pertain to PWA [39] with examples provided [40]. Shadden et al. [39] prefer the term “self” over “identity,” viewing “self” as a more holistic and personal concept, described as “a personal project.” They associate identity primarily with the dimension of roles [39].

Pound et al. [38] explore theory of identity and therapeutic approaches focused on promoting personal growth, self-exploration and self-actualization. They categorize identity into three types: personal, social, and collective. Additionally, Pound et al. [38] highlight how unfamiliarity with aphasia in society poses a barrier for PWA in accessing services.

#### Theoretical papers

The five theoretical papers present the work of Shadden and colleagues [2,41–44]. Four of these papers [41–44], ranging in dates from 2004 to 2007, mirror the content found in the book chapters described in the previous paragraph. In these chapters, the theories and definitions of identity used in the four theoretical papers appear to result in the Four-Domain Interdisciplinary Framework of the narrative self [8]. The topics discussed in the papers are: identity related to aphasia and the impact on life participation and integration in the Life Participation Approach to Aphasia (LPAA), as well as a review of concepts from sociocultural literature in order to understand the consequences of aphasia and the impact of aphasia on storytelling and self.

In the first paper, Shadden and Agan [41] provide an overview of varied interventions addressing identity issues. In the second paper, Shadden [42] refers to theory of Taylor [45] to define identity, and the impact of aphasia on identity is discussed. In the third paper Shadden and Hagstrom [43] discuss different concepts and theories found within psychological, communication, and sociological literature to understand narrative identity. Among others, they incorporated Holstein and Gubrium’s [46] theory of self, McAdams [47] theory of narrative identity, and Frank’s theory of narrative types [48]. In the fourth paper Shadden and Koski [44] synthesize various theories to understand the impact of aphasia on storytelling and self, employing Holstein and Gubrium’s [46] theory of self and Giddens’ [49] theory of self-identity. Finally, the fifth paper by Strong and Shadden [2], published in 2020, provides an overview of available narrative interventions and utilizes narrative theories from Taylor [45], McAdams [47], Bamberg [50], and Bruner [51].

#### Empirical papers focused on identity changes

Among the 12 empirical papers, three were specifically dedicated to exploring identity changes in aphasia. These papers range in dates from 2015 to 2020. Musser et al. [52] conducted interviews with 24 individuals affected by aphasia following a stroke, utilizing a grounded theory approach. Taubner et al. [9] investigated identity shaping in nine working-age people with chronic aphasia in a digitalized society. They incorporated Giddens’s [49] theory of self-identity along with Bamberg’s dilemmatic-space theory [50] to understand the concept of identity. Their methodology included both interviews and participant observation. Guerrero-Arias et al. [53] conducted a case study on the intersectional identities of a participant (a black, middle-aged, Colombian woman of low socio-economic status) and how these related to social interaction. They employed Norton’s social identity theory [54] and Crenshaw’s intersectionality theory [55] as frameworks to analyze the concept of identity.

#### Empirical papers focused on identity interventions

Five studies were specifically focused on identity interventions. These ranging in dates from 2010 to 2018. Simmons-Mackie and Elman [56] employed discourse analysis to pinpoint significant interactions during a group aphasia therapy session. They drew on Shadden and Agan [41] to define identity, and on Pound et al. [57] for theory of identity, differentiating between personal, social, and collective identity.

Bronken et al. [58] explored experiences of PWA who participated in an intervention aimed at supporting the process of psychosocial adjustment and enhancing psychosocial well-being. They drew on narrative theory from Frank [48], Bruner [51], and Polkinghorne [59]. The intervention was based on Zoffmann’s [60] “Guided Self-Determination” principles, and structured as an individual, dialogue-based collaborative process. The contents addressed different psychosocial issues such as mood, social relationships, meaningful activities, identity, and body changes. Employing a longitudinal, complex health intervention development design, the study incorporated participant observation, semi-structured interviews, and standardized clinical assessments involving seven individuals in the subacute stage of aphasia.

Bronken et al. [61] utilized participant observation and semi-structured interviews. Their aim was to elucidate the interactive process of narrative co-construction between a young woman with aphasia and a nurse during the first year after stroke, as part of a longitudinal psychosocial intervention. The intervention was based on Zoffman’s [60] “Guided Self-Determination” principles. Bronken et al. [61] employed the narrative theories of Frank [48], Bruner [51], Polkinghorne [59], and Ricoeur [62]. Additionally, they used Holstein and Gubrium’s [46] theory of self, and Bury’s [63] theory of biographical disruption in their report.

Corsten et al. [15] evaluated and adapted an interdisciplinary biographic-narrative intervention, and investigated how this intervention influenced identity negotiation and quality of life in aphasia using a mixed-methods design. They incorporated McAdams [47] and Bauer et al.’s [64] narrative theory, and Bury’s [63] theory of biographical disruption within their theoretical framework. Corsten et al. [15] employed quantitative measures of Health-Related Quality of Life (HRQL) in 27 PWA, and they conducted interviews to gain a deeper understanding of identity formation in PWA.

Lastly, Strong et al. [65] explored experiences of PWA who had participated in the My Story project, aimed at co-constructing personal narratives about living with aphasia. They utilized Taylor’s [45] definition of identity and Ricoeur’s [4,62,66] and McAdams’ [47] theory of narrative identity within their theoretical framework. Strong et al. [65] used a phenomenological design, conducting semi-structured interviews in three middle-aged men with chronic aphasia.

#### Empirical papers focused on narratives

The remaining four empirical papers represented reports, ranging in dates from 2006 to 2012, that were not specifically designed to investigate identity in PWA. Instead, they utilized a narrative approach to explore lived experiences of PWA. These studies were incorporated into this review due to the interrelationship of narrativity and identity [2,15], with identity issues emerging as outcomes elaborated upon in the discussion.

Hinckley [67] conducted a review encompassing twenty published accounts authored by PWA, available in books and peer-reviewed journals. The objective was to discern the elements contributing to successful living with aphasia post-stroke. In the review, Hinckley [67] employed Frank’s [48] theory of narrative types and Bury’s [63] theory of biographical disruption.

Barrow [68] conducted a case study examining the role of disability narratives in a middle-aged woman with chronic aphasia and her proxies to understand their understanding of stroke and aphasia. Barrow incorporated Frank’s [48] theory of narrative types, Somers’ [69] narrative theory, as well as Corker and French’s [70] theory on the narrative voices of disability in her work.

Mitchell et al. [71] explored various narrative types and metaphors employed by eleven PWA. They integrated Frank’s [48] theory of narrative types, Bury’s [63] concept of biographical disruption, and Ricoeur’s [4] theory of narrative identity within their theoretical framework to gain deeper insights into the narratives.

Finally, Armstrong et al. [72] used a narrative approach to understand how Aboriginal Australian people adjusted to aphasia over time, interviewing three middle-aged males in the chronic stage of aphasia. Armstrong et al. [72] underscored the importance of a holistic and narrative approach to understanding the experiences and stories of people with disabilities across diverse cultures, drawing on Hinckley [73].

### Descriptive summary of the reports

The empirical papers comprise a range of different settings and population characteristics as shown in Table A1. Most research took place in the chronic phase of aphasia (> 6 months post onset). Of the 12 empirical studies, 95 participants were recruited through speech-language pathologists, from ambulant rehabilitation and aphasia support groups or advertising *via* an aphasia association. In three studies, participants were included in the rehabilitation phase (from >1 ≤6 months post onset). These participants joined an intervention program in a hospital [52,58,61]. As for the review, only personal narratives (co-)authored by PWA that had been published in a book or professional journal were included [67]. Often it was unclear whether people lived independently at home or were resident in a nursing home. Age varied from 30 up to 78 years old. In four studies, the focus was on middle-aged PWA [9,65,68,72], age ranging from 35 to 65 years [74]. Two case studies focused on young women [53,68]. In the other studies, life stage and age were not considered or not substantiated. Additionally, there was a discrepancy between the proportion of men and women who were included in the studies; 31 women were included against 64 men. Gender was not substantiated for in the review [67]. Furthermore, most studies were conducted in Western countries, mostly in the USA. One study was carried out in Colombia [53]. In this study, social economic status and cultural background were considered. Information on social economic status was provided in one other study [52]. Cultural background was considered in an Australian study on Aboriginal PWA [72] and an Irish study [68].

### Perspectives on identity and identity formation

In this review the different approaches to the concept of identity used in studies on identity changes of PWA are analyzed. The following perspectives on identity and identity formation were based on the theoretical background on identity formation from all 20 included reports. The analysis revealed that various theories and definitions were used to describe identity. There was limited uniformity in the definition of identity in the articles. Thus, some authors refer to the “self,” others to “narrative self” [8] or “narrative identity” [15], while some distinguish between different types of identity such as “self-identity” [9], “personal identity,” “social identity,” “collective identity” [40,56], “chronotype,” and “intersectional identity” [53] or “occupational identity” [52]. Additionally, different synonyms are used for identity “formation” (e.g., “renegotiation,” “shaping,” “construction,” “adaptation,” “adjustment,” and “development”). Furthermore, identity is interpreted differently between disciplines. In the studies, many different authors and theories are cited to describe identity. This proves identity to be a complex concept which is challenging to define.

**Figure 2.**
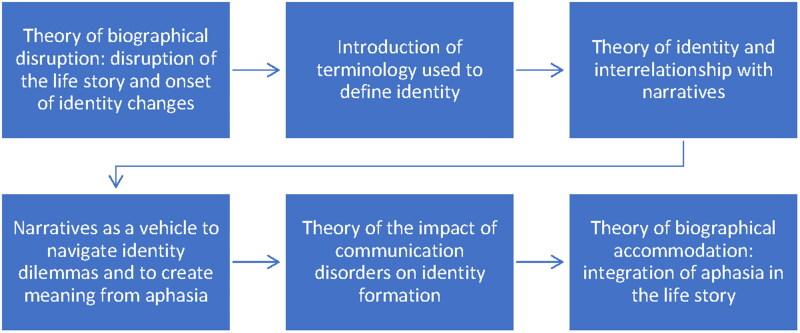
Step-by-step introduction of theory of the concept of identity in the reports.

However, there is also consistency in the theories cited and how they are used throughout the studies. In most studies, the concept of identity is explained in sequential steps (Figure 2). Explanation starts at the origins of post-stroke aphasia, that is, the biographical disruption theory introduced by Bury [63]. The semantic term used for identity in the article is then explained, and the concept of identity is discussed. Identity is clarified using the following theories: “narrative identity” [4,15,47,62,66], “narrative self” [8], “self-interpretation” [45], “self-identity” [49], “self-narrative” [51], “social identity” [54], “dilemmatic space” [50] and “intersectionality” [55]. Subsequently, the interrelationship between narrative, co-construction, and identity is elaborated. Most studies employ narrative theory from Ricoeur [4,62,66], and/or McAdams [47] for this purpose. Furthermore, theory of different types of narratives is discussed: “big and small stories approach,” i.e., “everyday narrative” versus “life story narrative” [2,50], and “illness narratives” as a vehicle to integrate aphasia into the life story narrative [48]. The impact of communicative difficulties of PWA on the construction of narratives and thus on identity formation is also discussed. Finally, the eventual goal of integrating aphasia into the life story and moving forward with one’s life, i.e., biographical accommodation, is explained [40].

The principal commonality between the theories used in the studies is that identity is considered a social construct, with storytelling as the navigating vehicle. Language and communication are an essential part of this process. Identity is negotiated in situational interactions in the present and evolves through time [4,39]. Life events are integrated into the life story, ensuring its continuity. Life story is influenced by one’s biographical characteristics i.e., gender, life stage, personality, physiology, and life history [39]. People have agency in how they present their identity in different social situations and roles. However, this does not mean that the self is always consciously created. The self is also formed as a result of our actions, rather than a conscious creation [39].

Another commonality in the studies is PWA’s struggle with societal perceptions of disability. PWA are faced with an abrupt change of their identity. Suddenly, they become “disabled” and are challenged by the cultural perspectives on illness and disability [38,40,53,68]. Often, cultural definitions of disability are associated with negative labels, i.e., stigmatization [38]. Common manifestations of stigma include perceptions of incompetence, dependence, perceived lack of agency and reduced social appropriateness [39]. Many PWA lack the agency, and power to negotiate and create an identity that is accepted by ­others [39].

A final commonality is that in all studies identity is described as dynamic and subject to change. However, the areas of tension and interrelationship between different aspects or dimensions of identity are not always reflected in the definitions used in the articles. It becomes clear from how identity is introduced in the literature that it is of interest to relate multiple perspectives. As mentioned earlier, identity seems to be best explained in sequential steps (Figure 2). This corresponds to the process of “maintaining and modifying life story and narrative self” [8] in which the following steps are represented: life story and narrative self, disruptive event, illness narratives, and biographical accommodation.

### Thematic summary of identity changes and challenges

**Figure 3.**
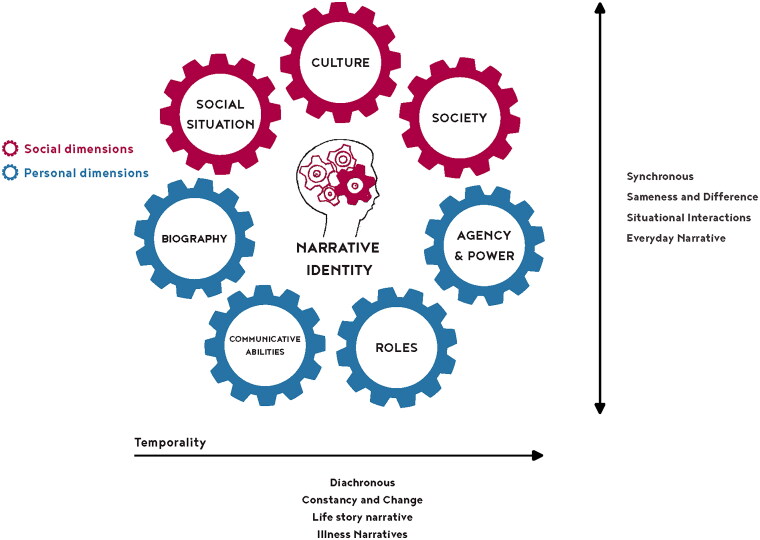
The “Narrative Identity Model.”

Within the studies, different research designs were used, and the results were analyzed by different disciplines from different perspectives. As a result, we found considerable variety in themes. These themes are systematically presented in Table A3 (Appendix A), which presents overarching themes, sub-themes and examples from the reports in a structured manner. However, this tabular representation lacks the representation of the dynamic and interwoven nature of identity, leaving the interrelationships between these themes concealed. A further step was taken in this regard by attempting to synthesize the aforementioned complementary theories into the “Narrative Identity Model” (Figure 3), to achieve an appropriate description of identity.

### The “Narrative Identity Model”

The “Narrative Identity Model” depicts:

1. Identity as a social construct with storytelling as the navigating vehicle and language and communication as an essential part of this process.
2. Dimensions and underlying concepts of the interdisciplinary four-domain framework of Shadden et al. [39].
3. Challenges that PWA experience while navigating their new identities based on Bamberg’s dilemmatic-space theory [50] and Ricoeur’s narrative identity theory [4,62,66].

The bottom of the model represents the “I” (personal dimensions) and the top represents the “We” (social dimensions). Narrative identity becomes visible in interaction with others at the intersection of the “I” and the “We” (illustrated by the head containing interconnected gears). The “I” refers to the personal dimensions: roles, communicative abilities, agency and power, and biography such as personal and physical characteristics and life story. The social dimensions refer to the social groups a person belongs to, culture e.g., norms, values, and semiotic signs (language and communication), and society or the world we belong to. The horizontal arrow represents temporality and refers to diachronic identity formation. The vertical arrow indicates synchronous identity formation in the present moment.

The “Narrative Identity Model” employs gears in different colors to represent themes related to narrative identity that interact. The themes are based on the analysis of the reports included in this review. The gears signify not only the interaction between the bottom “I”—personal dimensions, symbolized by blue gears—and the top “we”—social dimensions, symbolized by red gears—but also the interactions across various personal and social dimensions. This visualization underscores the dynamic interplay across dimensions, rather than depicting distinct selves.

The interactive relationship between the “I” and the “We” in the “Narrative Identity Model” is inspired by Ruijters et al.’s [75] “Professional Identity Model,” which is based on research outside the domain of aphasia. Ruijters et al. [75] distinguish between the personal, social, and collective self, using frames and half circles to depict interactions between the bottom half (“I”) and the top half (“We”). The themes in their model differ from those in the “Narrative Identity Model,” indicating that different themes influence the formation of narrative identity compared to professional identity.

### Overview of sub-themes in the Narrative Identity Model

**Figure 4.**
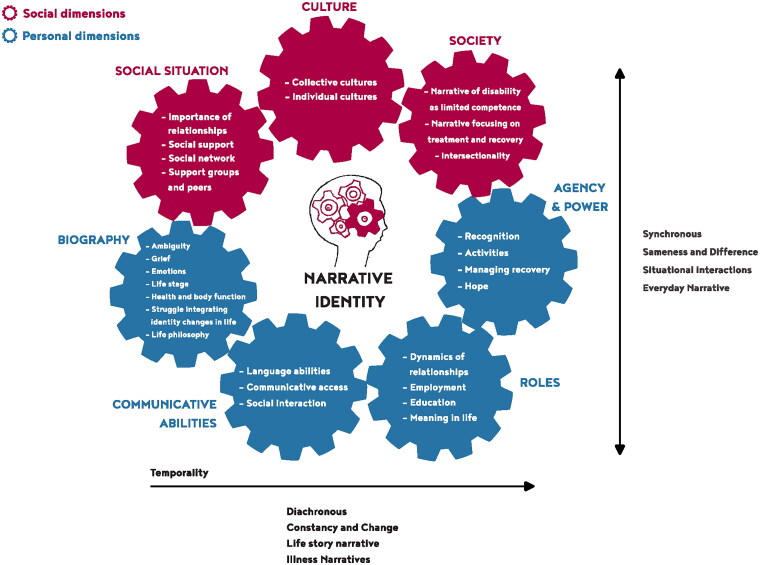
The sub-themes incorporated in the “Narrative Identity Model.”

The Narrative Identity Model attempts to visualize the dynamic nature of identity and the challenges PWA face while navigating their new identities. PWA experience inner variation within themselves. They consistently explain their experiences in relation to a broader context, which is why an experience can be labeled in different ways. Change can be both positive and negative, or cause sadness while simultaneously promoting growth. This reflects the ambiguous nature of living with aphasia [9,53,68] and demonstrates that one experience never excludes another. Recognizing the importance of addressing this ambiguity and the challenges PWA encounter in reshaping their identity, we incorporated the sub-themes in the “Narrative Identity Model” (Figure 4). This addition emphasizes how these themes interact and influence each other, highlighting the dynamic and multifaceted nature of narrative identity formation in PWA.

## Discussion

This scoping review aimed to provide an overview of existing knowledge on the concept of identity and identity changes and formation of PWA by exploring existing research on identity of PWA. In total, 20 reports were included, encompassing various types of reports (book chapters, theoretical papers, and empirical studies focused on identity changes, interventions, and narratives).

The findings revealed a range of different:

1. settings
2. population characteristics
3. frames and research designs to understand identity
4. theories and definitions to describe identity
5. themes regarding identity changes in PWA

The renowned framework of Arksey and O’Malley [32] was used to systematically conduct this scoping review.

### Theory of identity

In this review, various approaches to the concept of identity used in studies on identity changes in PWA were analyzed. Different terms and definitions were employed in the reports to describe identity. The analysis revealed that these approaches ultimately converged around a shared underlying construct, grounded in social constructionism as outlined in the introductory chapter. Whether authors refer to “self-identity,” “narrative identity,” or “social identity,” they all serve as lenses through which the socially constructed nature of identity in PWA is viewed.

Overall, the analysis of the reports provides several insights regarding identity changes in PWA. Following the onset of post-stroke aphasia, PWA experience biographical disruption and face identity changes [63]. Identity formation is a dynamic and interactive process where communication plays a crucial role, presenting additional challenges for PWA [2]. During the process of identity formation, PWA must navigate dilemmas both in situational interactions (synchronous) and through time (diachronous) [4,50,62,66]. PWA experience inner variation and ambiguity within themselves, implying that change can be both positive and negative simultaneously [9,53,68]. PWA require time to integrate aphasia into their lives (biographical accommodation), with their biography, communicative abilities, and agency and power influencing the process [39,40]. Aphasia profoundly impacts the roles individuals fulfill and meaning in life, as well as their social network. Moreover, societal perceptions of disability and cultural background significantly shape identity formation [53,68]. Recognition emerges as a fundamental need, yet PWA may lack agency to present themselves as they desire, potentially causing stigmatization [39]. The insights from the analysis are captured in the proposed “Narrative Identity Model,” which visualizes the interactive and dynamic nature of narrative identity formation among PWA, addressing its inherent ambiguity, the dilemmas faced, and the pivotal role of language.

Similar to Shadden et al. [39] we view identity as part of the self. Analoguos to Ruijter et al.’s [75] definition of professional identity, we consider narrative identity as that which inextricably identifies you as the person you are at the intersection of the personal and social dimensions. The head containing interconnected gears in the model serves as a reflection of one’s unique life story at this particular moment. Based on the analysis of the reports included in this review, we found, like Bamberg [50], that narrative identity emerges from the diachronic navigation of constancy and change and evolves through time. Moreover, we prefer identity formation above reconstruction or renegotiation. Besides the conscious nature of identity formation, this term also does justice to the unconscious part of the process.

### The search process

Conducting a search for literature on identity changes in aphasia was challenging due to the varied definitions of identity within the literature, a concept that encompasses many dimensions of daily life. We used multiple synonyms to ensure that all relevant publications on identity changes in aphasia were included. However, despite the rigorous search process, some studies may have been missed. In line with the aim of this scoping review, we opted not to examine the quality of the studies reviewed. Therefore, the results should be perceived as existing theories and themes that are important when considering the concept of identity and identity changes in aphasia.

### Population characteristics

Current research on identity changes in aphasia tends to focus on middle-aged PWA from Western cultural backgrounds. Also, more men than women are included in the studies and socioeconomic status is often not mentioned. Thus, little is known about the effects of intersectionality on identity formation of PWA, reflecting the marginalization of minority groups in both society and research. Moreover, most participants had received rehabilitation, were a member of an aphasia association, and/or were still attending therapy. This may have led to PWA, who have no access to therapy, being missed and may have affected the results.

In addition, the domestic situation of PWA was often not accounted for, even though it affects their position in the world and thus their navigation of agency to fit [50]. Notably, there is a lack of studies focusing on elderly PWA in nursing homes or those with early-onset aphasia, and how these conditions impact identity formation. Finally, most PWA were in the chronic phase, where aphasia might be more integrated into their life stories. This integration may result in aphasia being represented in their narratives in a different manner, influencing the reported experiences [72].

### Credibility

A major challenge for PWA regarding identity formation is their reduced language capacity, which complicates their ability to share narratives and reshape their identity. Furthermore, communication difficulties also hinder research on identity changes in PWA. Most studies rely on verbal data collection methods, such as interviews, during which communication was adapted to the needs of PWA. However, identity changes might be more comprehensively captured through a combination of verbal and nonverbal methods, such as the ethnographic studies conducted by Barrow [68], Taubner et al. [9] and Guerrero-Arias et al. [53]. This methodological triangulation allows for a more holistic examination of identity changes and formation, incorporating observations and the use of objects to enhance understanding.

### Interventions focused on identity formation

This review specifically addressed the concept of identity and themes related to identity changes and formation. The literature on interventions that focus on identity formation in PWA demonstrates that narrative interventions improve quality of life [15], well-being [58,61] and support a positive view of identity [65]. Additionally, narrative interventions contribute to an increased sense of competence and positive self-acknowledgement of PWA [15]. These findings show that appropriate treatment approaches are available to support identity formation of PWA.

Strong and Shadden’s [2] theoretical paper provides a clear up-to-date overview of available narrative interventions. No new or differing literature on narrative interventions and identity in PWA was identified in this scoping review. Therefore, we refer to their work for further details.

## Clinical implications and future research

This review demonstrates that identity formation is a complex and ongoing process, especially for PWA, who suffer from language disorders. Healthcare professionals should be aware of that and of the need for continuous attention to identity formation of PWA during rehabilitation.

Research on identity changes in PWA often employs verbal data collection methods. However, this review suggests that a combination of verbal and nonverbal methods could capture identity changes more fully. Triangulation of methods allows us to comprehend the multiplicity and complexity of experiences of PWA and how aphasia affects their life [76]. Additionally, the dynamics of how PWA’s narratives change over time in terms of identity has not been thoroughly explored. Therefore, we have initiated narrative inquiry combined with visual ethnography to longitudinally explore identity changes of PWA [77].

The review also highlights a significant gap in understanding the effects of intersectionality on identity formation in PWA. Therefore, healthcare professionals should be cautious when applying these findings generally, as they may not accurately represent every PWA. In addition, it is important to address different aspects of identity in future research to add to existing knowledge of identity changes and formation in PWA and to tailor future interventions if necessary. This can be done by including more PWA from non-Western backgrounds. Culture affects identity formation and this may be reflected in experiences of PWA. Furthermore, socioeconomic status, the domestic situation of PWA, life stage, and gender should be accounted for in the studies to do justice to experiences of a broader, more diverse group of PWA. Moreover, it is important to ensure that groups who are not known to receive speech therapy or do not have access to therapy are included in research. Also, the studies showed that the severity of communication impairment and the phase after the onset of post-stroke aphasia may influence experiences of PWA. Therefore, it is important to take these into consideration in research.

Our focus was primarily on identity changes of PWA. We did not encounter any literature that explicitly addressed identity changes of significant others of PWA. However, they experience identity changes as well. Thus, it is valuable to explore their experiences in future research.

Finally, in this review we decided not to include personal narratives of PWA themselves, as these cannot be considered scientific literature. Consequently, the results are influenced by the interpretations of different researchers, meaning that an important unfiltered perspective—that of PWA themselves—was overlooked. One way to do justice to this important viewpoint could be to include PWA as a co-researcher in research on identity changes of PWA.

## Conclusion

Our review found that the “Narrative Identity Model” could be used to clarify the concept of identity, since it integrates different theories of identity referred to in the literature on identity changes of PWA. Also, the model could be utilized as a tool to articulate the complexities and ambiguities inherent in identity changes. To date, research on identity changes in PWA has been limited, primarily due to the communication challenges they face, because their communication difficulties form an obstacle in research. Nevertheless, literature shows that addressing identity changes in aphasia is possible, especially when diverse research methodologies are integrated. In addition, growing evidence suggests that narrative interventions are effective in supporting identity formation and positively affect the quality of life of PWA. It is important for healthcare professionals to deepen their understanding of the identity changes experienced by PWA and to explore ways to support these changes in interdisciplinary settings, and enhance healthcare.

## Appendix A

**Table A1.** Descriptive summary of the reports.

Author, year & country	Aim of the study	Design and methods	Study population (age, gender, months post onset)	Findings of the empirical papers
Shadden, B.B.; Agan, J.P., 2004, USA [41]	To discuss identity as it relates to aphasia and the resulting impact on life participation.	Theoretical paper	N/A	N/A
Shadden, B.B., 2005, USA [42]	To review concepts from the sociocultural literature that bear upon social identity and can be applied to an understanding of short- and long-term consequences of aphasia	Theoretical paper	N/A	N/A
Hinckley, J.J., 2006, USA [67]	The question “What does it take to live successfully with stroke and aphasia?” was posed and answers were sought within already published accounts written by people living with aphasia.	Narrative design Review of personal narratives of PWA	*N* = 20, gender not specified, age 20s to 50s, ≥ 24 m.p.o.	The review showed four prominent themes associated with successful living with aphasia: the significance of social support, adaptations in self-perception, forward-looking attitudes with the establishment of new goals, and the agency for continual improvement in communication.
Shadden, B B.; Hagstrom, F., 2007, USA [43]	To explore the relationship and integration of a narrative and the life participation approach to aphasia (LPAA).	Theoretical paper	N/A	N/A
Shadden, B.B.; Koski, P. R., 2007, USA [44]	To address the impact of aphasia on construction or storying on self, rather than identity perse.	Theoretical paper	N/A	N/A
Barrow, R., 2008, Ireland [68]	To explore and identify the role that narratives of disability (i.e., the “inner stories we live by”) play in how Anne, a woman with aphasia, and those close to her, make sense of stroke and aphasia.	Single case study Narrative and ethnographic design In-depth interviews, participant observation, and collection of relevant artefacts	*N* = 1, female, age 40, 24 m.p.o.	The study showed two constant narratives in society: the narrative of disability as limited competence and the restitution narrative focusing on treatment and recovery. The narrative of disability as limited competence causes PWA to feel less valued, because they have to deal with the prejudice of others (e.g., concerning their perceived intelligence and abilities). The restitution narrative might give PWA the notion that the disability is not desired.
Shadden, B.B.; Hagstrom, F.; Koski, P. R., 2008, USA [39]	To explore the fundamentally social nature of the self by examining four primary dimensions - the cultural aspect, roles, situational interactions, and biography. To discuss the concepts of agency and power, which underlie all self-creation.	Book published by academic publisher, Chapter 3	N/A	N/A
Shadden, B.B.; Hagstrom, F.; Koski, P.R., 2008, USA [40]	To introduce a broad spectrum of individuals -male and female, married and divorced, retired and working at the time of stroke - with a variety of occupations and interests. They serve as an illustration of our focus on how people with communication disorders create narrative selves.	Book published by academic publisher, Chapter 8	N/A	N/A
Simmons-Mackie, N.; Elman, R.J., 2010, USA [56]	To describe identity relevant interactions in a group aphasia therapy session and identify explanatory themes associated with positive identity negotiation in order to contribute to the process of unpacking identity negotiation as an interactive phenomenon in aphasia.	Discourse design Video-taped group therapy session	*N* = 7 men, *N* = 1 female, group leader, group assistant, age 40–70 years, > 6 m.p.o.	The study showed five key themes linked to fostering a positive identity through interaction: being heard, acknowledgment of competence, solidarity, visualization of group identity markers, and the promotion of positive personal identity. These findings suggest the potential for incorporating identity-enhancing interactions as an integral component of aphasia therapy.
Mitchell, K; Skirton, H.; Monrouxe, L., 2011, UK [71]	The aim of this study was to explore the narrative types and metaphors used by people with aphasia. The objectives were to: (1) investigate the different narrative types and plot lines used; (2) explore the links between their use of narrative types and metaphors; and (3) suggest possible implications of the findings for reconstructing self-identities.	Narrative design Open and semi-structured interviews	*N* = 7 men, *N* = 4 women, age 49–78 years, 2,5 up to 10 years p.o.	The study showed four distinctive narrative types: amelioration, discordant, regeneration, and acquiescent. Furthermore, thirteen metaphorical concepts across five domains were identified: life, brain, spirituality, progress, and aphasia.
Armstrong, E.; Hersh, D.; Hayward C.; Fraser, J.; Brown, M., 2012, Australia [72]	To find out how aphasia was constructed and dealt with in Aboriginal communities and try to understand more about how Aboriginal people adjusted to aphasia in the long-term.	Narrative design Semi-structured interviews	*N* = 3 male, age 47–63, 6 months up to 29 years p.o.	The study showed a lack of discussion of aphasia in the narratives of the participants. In collective cultures, disabilities are construed differently compared to Western cultures, which tend to be more individualistic. This variance in cultural perspectives might influence how aphasia is perceived, discussed, and managed, consequently shaping the significance of aphasia and the position it holds in the lives of PWA.
Bronken, B.A.; Kirkevold, M.; Martinsen, R.; Wyller, T.B.; Kvigne, K., 2012, Norway [58]	To explore how seven persons with aphasia experienced participating in a complex nursing intervention aimed at supporting the psychosocial adjustment process and promoting psychosocial well-being.	Complex health intervention development design, intervention study Participant observation, semi-structured interviews and standardized clinical instruments	*N* = 1 female, *N* = 6 male, age 33–72, 1–3 m.p.o.	The study showed several aspects appreciated by participants: opportunities for dialogue, enhanced confidence in expressing thoughts and feelings, motivation to persevere through the challenging recovery process, and an evaluation of their life situation as relatively positive, despite encountering both challenges and fluctuations. These aspects confirm the meaningfulness of a collaborative narrative process in addition to psychological support and motivation to move forward during the challenging adjustment process.
Bronken, B.A.; Kirkevold, M.; Martinsen, R.; Kvigne, K., 2012, Norway [61]	To illuminate how an interactive process of coconstructing stories was established between a person with aphasia and a nurse within the context of a longitudinal psychosocial intervention during the first year after stroke.	Single case, intervention study Phenomenological design Participant observation and semi-structured interviews	*N* = 1 female with aphasia, age 30, 1 m.p.o., *N* = 1 female nurse, age 50	The study showed an improvement in well-being with three underlying mechanisms emerging: the need to tell, opportunity to tell, and help telling.
Corsten, S.; Schimpf, E.J.; Konradi, J.; Keilmann, A.; Hardering, F., 2015, Germany [15]	To evaluate an adapted interdisciplinary biographic–narrative intervention using quantitative measures of health-related quality of life (HRQL) and mood. Additionally, semi-structured interviews were conducted to gain a deeper understanding of identity development processes in people with aphasia.	Mixed methods, intervention study Grounded theory design In-depth interviews and standardized clinical instruments	*N* = 15 male, *N* = 12 female, age 44–73, 6,5–208 m.p.o.	The study showed a significant growth of HRQL and improved mood from participants’ self-reports. Four identity issues emerged from the analysis: agency, control, disease concept, and doing things.
Musser, B.; Wilkinson, J.; Gilbert, T.; Bokhour, B.G., 2015, USA [52]	A qualitative study with the aim of understanding the lived experience of individuals with aphasia and the trajectory of the effect on their social identities, from primarily their own perspectives (and including input and amplification by their partners) in the months and years after their stroke.	Grounded theory design Semi-structured interviews	Cohort 1: *N* = 2 women, *N* = 10 men, Cohort 2: *N* = 2 women, *N* = 10 men, age 46–71 years old, cohort 1 recently experienced a stroke to 18 m.p.o., cohort 2 > 5 years p.o.	The study showed alterations in three identity aspects: occupational, family, and social aspects.
Pound, C.; Parr, S.; Lindsay, J.; Woolf, C., 2018, UK [38]	This book focuses explicitly on therapeutic techniques developed from a social model approach to disability and learning to live with difference. It describes theories, activities and methods of implementation developed from the work of Connect with people with long term aphasia.	Book published by academic publisher, chapter 5	N/A	N/A
Strong, K.A.; Lagerwey, M.D.; Shadden, B.B., 2018, USA [65]	To examine the experiences of persons who had engaged in a project to co-construct personal narratives about life with aphasia.	Phenomenological design, intervention study Semi-structured interviews and standardized clinical instrument	*N* = 3 men, age 55–66 years, 4–5 years post onset	The study showed three overarching themes that participants raised after engaging in the project: more than a story, a positive experience and hope. The co-construction process contributed to fostering a positive perspective on identity and empowerment, enhancing confidence both in communicative abilities and self-perception. These themes affirm the meaningfulness of participants’ engagement in co-constructing personal narratives concerning their experiences of living with aphasia.
Guerrero-Arias, B.E.; Agudelo-Orozco, A;. A. Pava-Ripoll, 2020, Colombia [53]	How does Margarita’s intersectional identities relate to social interaction?	Single case study Intersectional identity chronotopes design Participant observation and in-depth interviews	*N* = 1 female, age 31, 12 m.p.o.	The study showed that societal perceptions restricted identity development, agency, and aspirations, particularly as a black woman navigating intersecting oppressions.
Strong, K.A.; Shadden, B.B., 2020, USA [2]	To provide an overview of the relationship between narrative, identity, and social co-construction for persons with aphasia and of narrative treatment approaches targeting identity renegotiation. The intent is to provide speech-language pathologists (SLPs) with background on how these key concepts fit within the Life Participation Approach to Aphasia and to empower them to consider engaging in personal narrative co-construction with their clients.	Theoretical paper	N/A	N/A
Taubner, H.; Hallén, M.; Wengelin, A, 2020, Sweden [9]	To investigate self-identity construction, in terms of identity dilemmas, in working-age persons living with post-stroke aphasia in a digitalised society (i.e., Sweden). Are the dilemmas relevant to the participants, and if so, how do they navigate them online and offline?	Multi method qualitative design Problem centered interviews and participant observations (netnography)	*N* = 3 men, *N* = 6 women, age 29–61 years, 13–96 m.p.o.	The study showed that all three dilemmas of Bamberg’s [50] dilemmatic space theory are interrelated and of high relevance to the participants. Diverse environments, offline or online, require different ways to navigate the dilemmas. The internet provides a platform to tell stories of self that differ from those offline. The results showed that online narratives not only reflect pre-stroke identity, but also align with expected social behavior in current online settings.

**Table A2.** Descriptive summary of theory of the reports.

Theoretical premises and definitions of identity	Cited by
Cited theories from external scholars with brief explanations	
Taylor [45,p.33] defines identity as “who we are, and where we are coming from,” which underscores the value of the concept of narrative identity.	Strong and Shadden [2] Shadden [42] Bronken et al. [58] Strong et al. [65]
Holstein and Gubrium [46] state that the personal self unfolds in social situations. As we engage in social interactions, the personal self forms as the central narrative through which we express our identity.	Shadden et al. [40] Shadden and Hagstrom [43] Shadden and Koski [44] Bronken et al. [61]
McAdams [47] suggests that individuals construct their sense of self by narrating personal stories to themselves and others, shaping the meaning of their lives.	Strong and Shadden [2] Corsten et al. [15] Shadden et al. [39,40] Shadden and Hagstrom [43] Bronken et al. [58]
Frank [48] proposes three types of illness narratives. Quest narratives in which illness is perceived as a journey in which meaning is sought. Restitution narratives focused on the restoration of health. Chaos narratives that depict a loss of control or disruption.	Pound et al. [38,57] Shadden et al. [40] Shadden and Hagstrom [43] Bronken et al. [58] Bronken et al. [61] Strong et al. [65] Hinckley [67] Barrow [68] Mitchell et al. [71]
Giddens [49] defines self-identity as the identity formed through an individual’s reflexive understanding of their own life story (biography).	Taubner et al. [9] Shadden et al. [39,40] Shadden and Koski [44]
Bamberg [50] proposes the identification of three practical challenges that self and identity formation processes are facing. These challenges are explained in dilemmatic spaces in which identity activities are narrated. The three dilemmas are: Successful diachronic navigation between constancy and change: “Becoming who I am” through time Creation of a synchronic connection between sameness and difference: “Who am I?” with regard to others Navigation of agency between the double-headed arrow of a person-to-world versus a world-to-person fit	Strong and Shadden [2] Taubner et al. [9]
Bruner [51] suggests that the relationship between life and narrative is reciprocal, both shaped through human rational processes. He argues that recounting one’s life is a cognitive achievement and fundamentally an interpretive act.	Strong and Shadden [2] Shadden et al. [40] Bronken et al. [58]
Norton [54] views identity as contradictory, dynamic and diverse, or “how a person understands his or her relationship to the world, how that relationship is constructed across time and space, and how the person understands possibilities for the future” [54,p.5].	Guerrero-Arias et al. [53]
Crenshaw’s [55] intersectional identity theory refers to the complex and interconnected nature of an individual’s identity, which encompasses multiple aspects, such as race, gender, class, sexuality and disability. Rather than considering each of these aspects separately, intersectionality emphasizes how these aspects of identity intersect and interact, shaping a person’s experiences, opportunities, and societal perceptions.	Guerrero-Arias et al. [53]
Polkinghorne [59] regards storytelling as fundamental to the continuous process of creating meaning and to self-understanding. Polkinghorne suggests that an individual’s narrative comprehension of their own life influences behavior that expresses this personal narrative. Language serves as a crucial tool for human interaction, social engagement, and community involvement. It is through language that we interpret and understand life events and experiences.	Bronken et al. [58] Bronken et al. [61]
Zoffmann’s [61] Guided Self-Determination approach supports participants gain new life skills to cope with the psychosocial impacts of stroke. The approach is based on principles of empowerment and emphasizes the significance of individuals taking charge of their own adaptation processes. Within this approach, the function of health care professionals is envisioned as that of a "supporter" or "coach," rather than that of a traditional "caregiver" or "therapist.	Bronken et al. [58] Bronken et al. [61]
Bury’s [63] biographical disruption theory refers to the effects of a major, unexpected event that profoundly alters the life story. From this perspective, chronic illness, such as aphasia, is considered as a disruptive event.	Corsten et al. [15] Shadden et al. [39] Pound et al. [38,57] Bronken et al. [58] Bronken et al. [61] Hinckley [67] Mitchell et al. [71]
Bauer et al.’s [64] state that identity is formed through the creation of life narratives in social interaction. Biographic self-thematization is seen as a continuous, collaborative process of making sense of experiences, using language as the primary medium. This process links the past, present, and future into a coherent storyline that gives life unity, purpose, and meaning, which are essential for mental health. Furthermore, the concept of quality of life is tied to finding meaning in life, highlighting the critical role of narrative identity in influencing quality of life.	Corsten et al. [15] Shadden et al. [40]
According to Ricoeur [4,62,66], people construct an understanding of who they are by giving meaning to the stories they exchange with others. This is what he calls narrative identity. Narrative identity is formed relationally and situationally in the present (synchronic) and evolves through time (diachronic).	Shadden et al. [39] Bronken et al. [61] Strong et al. [65] Mitchell et al. [71]
Somers [69] describes narratives as “stories one lives by” which are rooted in our beliefs, values, and worldviews, developed gradually and shaped by our personal, societal, and cultural circumstances. Individuals interpret disruptions by attempting to incorporate or reconcile them within these narratives.	Barrow [68]
Corker and French [70] state that individuals are exposed to a variety of complex narrative voices concerning disability, which shape their interpretation and understanding of what disability means. Within the social model approach, it is now recognized that the impacts of the impairment itself also play a role in shaping the experience of disability.	Barrow [68]
Hinckley [73] suggests that acknowledging identity through a narrative-based approach offers a holistic view. She emphasizes that the personal, social, and cultural environments of individuals living with communicative disabilities should be central to the research and clinical strategies of speech-language pathologists.	Armstrong et al. [72]
Original theories and definitions from reviewed reports with brief explanations	
Pound et al. [38, 57] identify three types of identity. Personal identity involves the formation and consolidation of an individual’s sense of self, the personal biography. Social identity pertains to one’s inclusion in (or exclusion from) different social and cultural groups, communities, and institutions [78]. Collective identity focuses on a group’s sense of unity, equality, and purpose, typically characteristic of political and advocacy groups, where individual issues are subordinate to a certain extent.	Simmons-Mackie et al. [56]
Shadden et al. [39] designed the four-domain interdisciplinary framework of the narrative self to provide foundation for discussion of the narrative self in neurogenic communication disorders. This framework addresses four different dimensions of everyday life (cultural aspect, roles, situational interactions, biography) and underlying concepts out of which the self is constructed (agency and power) in order to discuss the narrative self.	N/A
Shadden and Agan [41] define identity as “a composite of roles, values, and beliefs that are acquired and maintained through social interaction” [38,p.175].	Simmons-Mackie et al. [56]
Corsten et al. define narrative identity as “the internal, dynamic life story that is constructed in order to create sense and meaningfulness” [15,p.796].	N/A

**Table A3.** Overview of (sub-)themes of identity changes in aphasia.

Themes	Sub-themes, originating from the analysis, illustrative examples and their sources			
*Communicative abilities*	*Language abilities* Reduced language abilities [9,38,41–44,52,53,58,61,65,67,68,71,72] Loss of fluency in language timing and pace [9] Tiredness [40,58] Difficulties with concentration, understanding [58], and self-expression [40,58] Frustration when partner does not adjust communication appropriately [40]	*Communicative access* Reduced access contacting authorities, handling administration, participating in social situations, performing their jobs, and pursuing studies Social barriers [9,52,53,58] Lack of public awareness [38,40]	*Social interaction* Difficulty sharing their story [2,40,53], and engaging with others [40,52,58] Feeling stupid or boring in interaction [9] Difficulty participating in arguments and discussions [53] Withdrawing from and enjoying social situations less [40,52] Being less able to frame experiences in adaptive ways through language [52]	
*Society & culture*	*Narrative of disability as limited competence* Split between “non-disabled” and those labeled “disabled” [71] Close relationship between aphasia and the concept of competence [38,40,53,68,71] Prejudice [9,68,71] Stigmatization [38,40,53,68] Assumptions of what characterizes successful individuals [38,40,68]	*Narrative focusing on treatment and recovery* Working hard and having faith will support recovery [9] Life revolves around activities that support improvement [68]	*Intersectionality* Being discriminated against because of ethnicity, gender or disability [53]	*Culture* Individual cultures in which communication problems and implications were explicitly reported [9,52,67,68,71] and PWA were interested to understand aphasia and brain function [71] Collective cultures in which disabilities are construed differently than in individual cultures [72]
*Social situation*	*Importance of relationships* Importance of grandchildren, family, friends [72], groups, and communities [40] Being needed gives the courage to continue [72]	*Social network* Changed social network [52,58] People disappeared [40,52,58] Divorce [40,68] Isolation [58]	*Social support* Social support is vital [9,52,67] Support received from unanticipated places [52] People not as helpful as expected [52]	*Support groups and peers* Support groups helpful environment to share stories and experiences about living with aphasia [40,41,56] Nice to meet people in the same situation [9,40] Feeling more comfortable to reveal and incorporate disability [9] Some become leaders and role models for their peers [9,52,71] Providing hope after stroke to other PWA [52,65]
*Biography*	*Grief* Loss of pre-stroke identity [9,38,40–44,53,61,65,67,68,71] Identity theft [40,42,71] *Emotions* Fear of not being themselves Failure [9] Frustration [9,72] Feeling overwhelmed by the change [52] Uselessness, sadness and confusion [72] *Ambiguity* Feeling distressed and at the same time feeling more relaxed than before [68] Difficulty accepting vs becoming a better person [9,40]	*Struggle integrating identity changes in life* Struggle with the realization not being the same person as before [9,40] Difficulty accepting vs becoming a better person [9,40] Suicidal thoughts [71,72]	*Life stage* Considered unfair by the young [40] The young feel different than peers older in age [9] The middle-aged fulfill many roles which causes considerable identity changes [40] *Life philosophy* Spirituality and religion [40]	*Health and body function* Significant health issues might cause aphasia being secondary to other conditions [72] Altered body function such as hemiparalysis, apraxia causes re-learning of everyday tasks and slower pace [9,53,72]
*Agency & power*	*Recognition* Looking for responsiveness of other people [43] Tensions between how PWA see themselves and how identity is perceived by others [9,40,53] Being treated as incompetent and docile [40] Feeling ridiculed [9] and not accepted as the person they have become [67] Perceived illness without actual condition [58] Feeling keen to show capability [72]	*Managing recovery* Reading the paper each day [72] Adult literacy, education, and writing skills training [67,72]	*Activities* Changes in social activities [15,52,58] Return to activity [15,52,67,71,72] Being capable, active, and finding different ways of self-expression [72]	*Hope* Restricted aspirations [53] Forward-looking attitudes with the establishment of new goals [67] There is life after stroke [61]
